# Progress in treatment of facial neuritis by acupuncture combined with medicine from the perspective of modern medicine: A review

**DOI:** 10.1097/MD.0000000000036751

**Published:** 2023-12-22

**Authors:** Qingxi Cao, Biao Qi, Lingyan Zhai

**Affiliations:** a Shiyan People’s Hospital of Baoan District, Shenzhen, China.

**Keywords:** acupuncture treatment, combination of acupuncture and medicine, facial neuritis

## Abstract

Facial neuritis is a common clinical disease with high incidence, also known as Bell palsy or idiopathic facial nerve paralysis, which is an acute onset of peripheral facial neuropathy. In modern medicine, there have been obstacles to the effective treatment of facial neuritis. At present, the clinical use of Western medicine treatment is also a summary of clinical experience, the reason is that the cause of facial neuritis is unknown. Facial neuritis belongs to the category of “facial paralysis” in traditional Chinese medicine. For thousands of years, Chinese medicine has accumulated a lot of relevant treatment experience in the process of diagnosis and treatment. At the same time, traditional Chinese medicine, acupuncture and the combination of acupuncture and medicine play an important role in the treatment of facial neuritis. This article discusses the treatment of facial neuritis with acupuncture combined with Chinese medicine, based on the research progress of modern medicine. In this review, we provide an overview of the effectiveness of acupuncture and medication combinations and facial neuritis with current studies investigating acupuncture and medication combinations in the treatment of facial neuritis.

## 1. Introduction

Facial neuritis is a peripheral facial paralysis caused by nonspecific inflammation of the internal facial nerve of the stylomilk foramen. The clinical manifestations are paralysis of the affected facial muscles, enlargement of the eye fissure, inability to close the eyelids, disappearance of the forehead lines, and ptosis of labial angle.^[1]^ At the same time, the epidemiological survey found that the incidence of facial neuritis was (11.5–55.3)/100,000 people. Usually, the symptoms of peripheral facial paralysis will be improved and cured within a few weeks or months, but sometimes it will lead to severe temporary oral dysfunction and eyelid closure dysfunction, which may cause permanent eye damage. About 25% of patients with facial neuritis may have persistent moderate to severe facial asymmetry, which has a very serious impact on the quality of life of patients.^[2]^ However, the etiology of facial neuritis is still unclear, and studies have found that it may be related to viral infection, inflammation, local trauma or cold, resulting in nerve inflammation, leading to focal edema, demyelination and ischemia. The treatment of acute facial neuritis is mainly based on symptomatic treatment such as hormone anti-inflammatory, nerve nutrition, antiviral, dehydration and detumescence, aiming at alleviating facial nerve edema, alleviating compression, controlling inflammation, nourishing nerve, improving local blood circulation, etc., so as to promote the recovery of facial nerve function.^[3,4]^ Western medicine treatment has certain limitations on the recovery of facial nerve in the early stage, and with the extension of medication time, adverse reactions are prone to occur, which makes patients suffer from psychological and physical pain, and is not conducive to the recovery of the disease.

In ancient China, the earliest description of facial neuritis can be found in the Miraculous Pivot of the Inner Canon of Huangdi: It is suggested that exogenous cold and heat are the main causes of facial neuritis. At the same time, Traditional Chinese Medicine (TCM) has a perfect understanding of the pathogenesis of facial neuritis, believing that in the early stage of facial neuritis, wind evil combined with cold, heat, dampness and other evil causes the blockage of meridians and collaterals, resulting in facial paralysis; in the middle stage, the blockage of meridians and collaterals lasts for a long time, and Qi and blood can not nourish facial muscles, resulting in muscle apraxia; Later, if facial neuritis is not treated in time, deficiency of Qi and blood will lead to contracture of meridians and tendons, even withering, and spasm. It is worth noting that there is no name of “facial neuritis” in traditional Chinese medicine, and modern physicians classify facial neuritis into traditional syndromes such as “mouth and eye deviation,” “facial paralysis,” “mouth deviation” and “hanging line wind” according to its clinical manifestations.

The basic theory of TCM treatment is “treatment according to syndrome differentiation,” in which discriminate is the pathological summary of judging the location, nature, severity and prognosis of a disease at a certain stage. According to the pathological results, certain treatment measures are given, such as traditional Chinese medicine prescription and acupuncture point selection. At the same time, in the thousands of years of development of traditional Chinese medicine, there is no unified conclusion on the etiology and pathogenesis of facial neuritis, but modern medical scientists have different opinions on the understanding of facial neuritis by integrating the research of Western medicine theory. At present, the treatment of facial neuritis with acupuncture and traditional Chinese medicine is mainly based on case reports and clinical trials, lacking of systematic review and summary.^[5]^ In this review, we analyze the current understanding of the combination of acupuncture and medicine and facial neuritis, and evaluate the current studies on the effectiveness of the combination of acupuncture and medicine in the treatment of facial neuritis.

## 2. Combination of acupuncture and medicine

Acupuncture and drug therapy has been applied since ancient times, and the organic combination of acupuncture and drug therapy shows the clinical effect characteristics of synergy and attenuation. Meridian system is the common effect pathway of drug therapy and acupuncture and moxibustion therapy. The application of meridian theory can organically combine acupuncture and moxibustion therapy and drug therapy in the diagnosis of disease location, evaluation of disease condition and prognosis, both internal and external treatment, with more obvious advantages. Acupuncture is one of the oldest medical practices in TCM and one of the most popular TCM treatments for thousands of years. Its safety and effectiveness are respected. Based on its superior safety and low side effects, it has been gradually recognized as a therapeutic intervention in complementary medicine.^[6]^ Meridians and acupoints represent complex structures, which have been confirmed by different potentials and histochemical compositions under the guidance of modern medicine. Traditional Chinese medicine believes that meridians and collaterals are important pathways connecting viscera and body surface. The nervous system of modern medicine is more accurate to connect the viscera with the body surface.^[7]^ Acupuncture and moxibustion is based on the application of certain stimulation to specific acupoints of the body to achieve the effect of treating diseases. The healing theory is derived from the understanding of the concepts of Qi and Yin and Yang in Traditional Chinese Medicine (TCM). Under the guidance of TCM, Qi is the “life energy” of human existence, and health is the normal alignment of Qi. The foundation of Qi theory is composed of 5 elements, including wood, fire, earth, metal, and water.^[8]^ Yin and Yang refer to the concept of balance of power in the material world, with Yin representing receptivity, tranquility, rain, coldness, and femininity, and Yang representing power, excess, daylight, heat, and masculinity.^[9]^ The purpose of acupuncture treatment is to realign the flow of qi through the body and balance the internal yin and Yang of the human body to reestablish harmony. Any blockage or excess will destroy the normal alignment of qi, and acupuncture can open the blockage or reduce the excess qi flowing through specific channels in the body (meridians) to restore it to its normal state, which is defined as “harmonizing yin and Yang” in traditional Chinese medicine theory. Yin and Yang represent the balance of the human body.^[6]^ At present, acupuncture is widely used to relieve a variety of pain diseases in the world and has been included in the guidelines. It is an effective way to deal with the opioid crisis. It is widely respected because of its safety, low price and low addiction.^[10]^ AEs associated with acupuncture often include subcutaneous hematoma, hemorrhage, skin bruising, and needle pain. Subcutaneous hematoma and bleeding at acupuncture points were the most common adverse events, but the risk factors of adverse events were related to the gender, age of patients and the local anatomical structure of acupoints. Improving the level of diagnosis and treatment of doctors can reduce and alleviate adverse events.^[11]^ At the same time, Liu et al^[12]^ introduced the anti-inflammatory mechanism of vagus-adrenal axis driven by electroacupuncture through animal experiments, which further illustrated the scientific nature of real acupuncture treatment. For Bell’s palsy, acupuncture and moxibustion treatment is believed to regulate the meridians and collaterals, regulate qi and blood, increase the excitability of nerves, promote the regeneration of nerve fibers and the formation of their collateral branches, enhance muscle contraction and blood circulation, and accelerate the recovery of metabolism and body functions.^[13]^

At the same time, drug treatment of facial neuritis is a commonly used recommended means in clinic, but in recent years, the literature on the treatment of diseases by the combination of acupuncture and medicine has found that the combination of acupuncture and medicine has better curative effect, and to a certain extent, it is better than the simple treatment plan, and the combination of acupuncture and medicine is a specific treatment method based on syndrome differentiation and treatment. In clinical practice, there are various forms of combination of acupuncture and medicine, in order to improve the ability of active use of acupuncture and medicine, and then improve the clinical efficacy.^[14,15]^ Acupuncture is the most commonly used rehabilitation treatment for facial neuritis; steroid hormone treatment for facial neuritis is a treatment method with good curative effect which has been repeatedly verified by modern medicine; Exercise therapy is the most effective method of modern rehabilitation medicine for functional recovery after nervous system damage. Xiao-hong Wang et al^[16]^ proved that the treatment of facial neuritis with integrated traditional Chinese and Western medicine has significant effect. For individualized treatment of facial neuritis, acupuncture combined with functional training and drug treatment can quickly eliminate clinical symptoms and signs. Improve the cure rate of patients and reduce the generation of sequelae. Therefore, the purpose of this study is to explore the advantages of the combination of acupuncture and medicine, and to provide new ideas for the diagnosis and treatment of facial neuritis.

## 3. Facial neuritis

### 3.1. Epidemiology

Facial neuritis is an acute attack of peripheral facial neuropathy, is one of the most common causes of lower motor neuron facial paralysis, also known as “unexplained acute facial paralysis,” is a common cranial neuropathy, resulting in unilateral facial muscle paralysis or complete paralysis, its main clinical features are unilateral lesions, sudden onset and usually progress within 48 hours. Studies have shown that about 70% of facial paralysis cases are related to Bell’s palsy, and the prevalence rate is (11.5–55.3)/100,000 people, and the prevalence rate is similar between men and women.^[2,17,18]^ Korean researchers found that the incidence of facial neuritis in Korea showed a rapid upward trend year by year from 2010 to 2018, and the study showed that the annual incidence per 100,000 people was 12.86% in 2010 and increased to 19.92% in 2018.^[19]^ At the same time, the study found that the incidence of facial neuritis was highly correlated with the age and season of patients. In the past 30 years, the study of facial neuritis showed that 12 studies reported the relationship between the prevalence of facial neuritis and age, and another 14 studies reported the relationship between season, weather and the incidence of facial neuritis. The results show that the incidence of facial neuritis is higher in the elderly, while the incidence of facial neuritis is related to low temperature and high pressure environment, and the incidence of facial neuritis is increasing due to the aging society and lifestyle changes.^[20]^ Specific risk factors for facial neuritis include a higher risk of facial neuritis in patients who are pregnant, have severe preeclampsia, obesity, hypertension, diabetes, and upper respiratory disease.^[21]^

### 3.2. Pathophysiology

Facial neuritis is a peripheral facial neuropathy in which the facial nerve receives axons from the solitary nucleus and the upper part of the superior salivary nucleus, containing a mixture of motor, sensory, and parasympathetic fibers; Facial neuritis is characterized by facial sensory changes (cranial nerve V), vestibular dysfunction (cranial nerve VIII), or pharyngeal symptoms (cranial nerves IX and X), because the facial nerve has intracranial, intratemporal, and extratemporal pathways, and forms a large number of connections with adjacent cranial nerves.^[22,23]^ Patients with facial neuritis were detected and evaluated by magnetic resonance imaging and neuroelectrophysiology, and their pathological changes were found to be due to facial nerve edema and bioelectric conduction disorders^.[24,25]^While the pathophysiology of facial neuritis remains unclear, the prevailing hypothesis is reactivation of the herpes simplex virus type 1 (HSV1) virus in the geniculate ganglion, leading to nerve edema and its compression through the petrous bone. HSV1 targets peripheral neurons and can be latent in the autonomic and sensory ganglia of the brain and neck of the host. It has a lifelong risk of infection and transmission and is the most common viral infection worldwide. However, viral infection was not detected in some patients with facial neuritis, so the clinical evidence that facial neuritis is caused by HSV1 infection is not sufficient.^[2]^ In the autoimmune hypothesis, facial neuritis is considered to be a single neuritis variant of Guillain-Barre syndrome. Studies have found that the decrease of T suppressor cells and the increase of B lymphocytes in patients with facial neuritis, as well as the increase of serum chemokine concentrations, are evidence that there is a cell-mediated immune mechanism in facial neuritis.^[26–29]^ At the same time, the inflammatory response of the body is considered to be an independent pathogenic factor of facial neuritis. The ratio of neutrophils to lymphocytes (NLR) is one of the markers of inflammatory response, which reflects the inflammatory and immune status of the body. Recent studies have detected higher levels of NLR in patients with facial neuritis, and there is a positive correlation between NLR and House-Brackmann (H-B) grade.^[30]^ Recent studies suggest that cold stimulation response may be an independent pathogenic factor of facial neuritis. Animal experiments have found that cold stimulation response can lead to cell pathway and gene dysfunction, thereby affecting the conduction of facial nerve signals, which may be the key factor of facial neuritis caused by cold stimulation response.^[31]^ a retrospective study in South Korea assessed the impact of meteorological factors on the onset of facial neuritis, and the results showed that the onset of facial neuritis was related to low temperature, low humidity and strong wind speed; a survey on the predisposing factors of facial neuritis in China showed that among 262 patients, 139 patients had a clear history of cold before onset, accounting for 53.5%.^[20,32]^ The pathogenesis of facial neuritis may be related to viral infection, facial nerve ischemia, inflammation and cold stimulation response, and further research on its pathophysiology is needed to get the exact cause of the disease, so as to promote the diagnosis and treatment of facial neuritis.

### 3.3. Diagnosis

The clinical manifestation of facial neuritis is the sudden weakness of facial expression muscles on one side of the face, which is usually noticed by patients when they look in the mirror or by family members, and drooling at the corners of the mouth may also be the initial symptom. Facial neuritis in patients with facial muscle weakness, often accompanied by pain behind the ear and the same side of the cheek paresthesia; rare will appear abnormal taste, the most rare will appear hyperacusis and other accompanying symptoms.^[33]^ Peripheral facial palsy can be clinically distinguished from central facial palsy (e.g., multiple sclerosis, stroke, tumor) by whether the forehead muscles are involved.^[34]^ Central nervous system lesions can also cause facial nerve paralysis. Supranuclear (central) lesions affecting the facial nerve do not paralyze the affected side of the forehead, leading to unilateral facial nerve paralysis, but the muscle function of the forehead is preserved.^[35]^ The typical features of facial neuritis are no wrinkles on the forehead, low eyebrows (drooping eyebrows), incomplete eyelid closure, drooping corners of the mouth, and flat nasolabial folds. Of course, incomplete (perioral) facial paralysis is difficult to distinguish from central facial paralysis in clinical manifestations, and other diagnostic measures are needed.

Facial neuritis belongs to peripheral facial paralysis, and its diagnosis often requires differential diagnosis with the following diseases. Ramsay-Hunt syndrome is caused by the activation of varicella-zoster virus in the geniculate ganglion. Its clinical manifestations include facial paralysis, hyperacusis, herpes in the external auditory canal, hearing loss and other symptoms, and 2% to 35% of patients are not accompanied by herpes zoster. Therefore, it is easy to be misdiagnosed as facial neuritis, which can be differentiated by varicella-zoster virus polymerase chain reaction.^[36]^ Lyme disease is an insect-borne infectious disease caused by Borrelia burgdorferi infection, which often occurs in summer and humid areas with high incidence, and most patients have a history of field activities. Facial paralysis is the most common neurological symptoms of the disease, most of which are bilateral involvement, most of which can recover and have a good prognosis. The history of outdoor activities and tick bites is very important for the diagnosis, and the diagnosis can also be made by the detection of anti-Borrelia burgdorferi antibodies in serum and cerebrospinal fluid.^[37,38]^ Human immunodeficiency virus (HIV) -related facial paralysis can occur in the early or late stages of HIV infection, and patients can have unilateral or bilateral facial paralysis, which can be differentiated from facial neuritis by HIV antibody testing.^[39]^ At the same time, studies have shown that 10% to 23% of facial paralysis is caused by trauma, and iatrogenic facial nerve injury is more common than accidental injury.^[40]^ At present, facial nerve magnetic resonance plain scan plus enhanced examination has been routinely used in the diagnosis of facial neuritis, and should be actively differentiated from the above diseases in clinical practice.^[41,42]^

### 3.4. Traditional treatment of facial neuritis

Facial neuritis is often divided into 3 stages in clinical research: acute stage, convalescence and sequelae. Acute period often comes on hind 1 to 2 weeks, convalescent period often comes on hind 2 weeks, sequela period comes on hind 3 months to half an year above commonly, according to facial neuritis the course stage that the patient is in and take different remedial method.^[43]^ At present, the treatment of facial neuritis is mainly drug therapy and non-drug therapy. Traditional treatment with oral corticosteroids has reduced facial nerve inflammation in patients with facial neuritis, and oral glucocorticoid therapy has been supported by high-quality evidence from a meta-analysis of randomized clinical trials. Glucocorticoid therapy has been found to improve the likelihood of recovery of facial nerve function, shorten recovery time, and reduce the severity of synkinesia and residual facial paralysis. However, corticosteroids can relieve clinical symptoms in the short term, but the long-term effect is not good, and there are many side effects and contraindications.^[44]^ Based on the hypothesis that HSV-1 may be a causative factor for facial neuritis, it is suggested that combination therapy with glucocorticoid drugs and antiviral drugs may be beneficial. However, it is still uncertain whether antiviral therapy can benefit patients with facial neuritis. Studies have shown that the combined antiviral and glucocorticoid treatment of mild facial neuritis patients has no significant benefit, but the combined treatment can increase the proportion of rehabilitation of facial paralysis symptoms in patients with severe facial neuritis.^[45]^ Therefore, antiviral treatment is recommended for patients with severe facial paralysis. Studies have found that valaciclovir is better than acyclovir in the recovery of facial nerve function.^[46]^ For other treatments, surgical decompression is recommended within 3 weeks of onset in patients with persistent loss of function (more than 90% loss of electroneurography) after 2 weeks. However, the most common complication of facial neuritis decompression surgery is postoperative hearing loss, which affects 3% to 15% of patients. The American Academy of Neurology does not currently recommend surgical decompression for facial neuritis because of the potential harm and the lack of data supporting benefit.^[47]^

### 3.5. Evidence for the treatment of facial paralysis by acupuncture combined with medicine

The etiology and pathogenesis of facial neuritis is relatively complex, and there is no final conclusion at present, and there is relatively little research on the etiology and pathogenesis of facial neuritis in traditional Chinese medicine. However, traditional Chinese medicine summarizes the pathogenesis of facial neuritis as follows: The lack of healthy qi, the empty vein, coupled with the invasion of wind evil, qi and blood obstruction, the loss of tendons and veins in the dysfunction of caring, muscle relaxation in constraints, resulting in the appearance of crooked mouth and eyes. Zhao Jiping’s modern clinical investigation shows that the onset of facial neuritis is affected by external factors such as cold, emotional fluctuation and fatigue, and the onset season is mostly in winter and autumn.^[48]^ The investigation systematically reflects that the pathogenesis of facial neuritis is characterized by deficiency of healthy qi and Wind chill causes disease. Traditional Chinese medicine treatment of facial neuritis has many methods, flexible clinical treatment, more accurate syndrome differentiation and treatment, can significantly improve symptoms, and reduce sequelae, so as to achieve the desired effect, it is worth promoting.^[49]^ Acupuncture has been shown to be effective and safe in several studies. Some systematic reviews have concluded that acupuncture is effective at the onset of facial neuritis.^[50]^ Modern clinical studies have found that when physical therapy (such as Kabat therapy, acupuncture and electrical stimulation) is added to drug therapy, the efficacy of drug plus physical therapy is significantly higher than that of steroids, placebo and antiviral drugs alone.^[51]^

In this section, we present and discuss various studies conducted for the treatment of facial neuritis with the combination of acupuncture and medication. Table 1 summarizes these studies. This review includes several recent randomized controlled trials (RCTs) and meta-analyses to evaluate the safety and efficacy of combined acupuncture and medication in the treatment of facial neuritis.

**Table 1:** Evidence for the treatment of facial neuritis with acupuncture combined with medication.

First author of the study (year)	Study groups and interventions	Results and findings	Conclusion
Ying Li et al (2004)^[52]^	A multicenter (4-hospital) randomized clinical trial of 439 patients with unilateral facial nerve palsy alone, ages 16 to 70 years, and onset of facial paralysis between 1 and 90 days, all patients were randomly assigned to a control group or 1 of 2 treatment groups. Interventions: The control group was treated with prednisone, vitamin B, vitamin B12 and dibazole; the treatment group 1 was treated with Acu-Moxi alone, and the treatment group 2 was treated with Acu-Moxi combined with the control group.	Based on the assessment of H-B scale and FDI score, the treatment effect of treatment group 1 and treatment group 2 was better than that of the control group after treatment, and there was a significant statistical difference.	This multicenter, single-blind, randomized controlled clinical trial provides support for Acu-Moxi and combined acupuncture and medication as an effective alternative treatment for patients with facial neuritis.
Fanrong liang et al (2006)^[53]^	Patients diagnosed with facial neuritis (n = 480) Intervention: The patients were randomly divided into 3 groups: control group (prednisone, vitamin B1, vitamin B12, bendazol treatment), acupuncture group (filiform needle plus moxibustion treatment), basic treatment plus acupuncture group (control group oral medicine plus acupuncture treatment).	The clinical efficacy of the acupuncture group and the basic treatment plus acupuncture group was better than that of the control group, with significant statistical difference. The H-B scores of the basic treatment plus acupuncture group and the acupuncture group were better than those of the control group (*P* < .01). The FDI scores of the basic treatment plus acupuncture group and the acupuncture group before and after treatment were better than those of the control group (*P* < .01).	Acupuncture treatment of Bell’s palsy is effective and can be used as an alternative to the treatment of facial neuritis.
Lian-Sheng Yang et al (2022)^[54]^	The clinical data of the Third Affiliated Hospital of Sun Yat-sen University from 2016 to 2021 were retrospectively collected. Interventions: All patients were treated with oral prednisolone. They were divided into 2 groups according to whether they had received acupuncture treatment within 7 days of paralysis onset.	The results of the study showed that patients who received acupuncture treatment within 7 days had a significantly shorter time to complete recovery, with a statistically significant difference. The recovery rate of the patients in the acupuncture group within 7 days was significantly higher than that of the patients in the non-acupuncture group within 7 days at the 12th week of treatment, with significant statistical difference.	This study shows that acupuncture treatment in the acute phase of facial neuritis can shorten the recovery time and improve the prognosis of patients with facial neuritis.
Qi-Hua Qi et al (2013)^[55]^	One hundred two patients with facial neuritis were randomly divided into electroacupuncture combined with acupoint application of Chinese medicine group (group A, 50 cases) and electroacupuncture group (group B, 52 cases). Interventions: Group A was treated with electroacupuncture combined with Yifeng acupoint application; Group B was treated with electroacupuncture.	The scores of facial nerve function in group A and group B were significantly improved compared with those before treatment, with significant statistical difference. The cure rate of group A was significantly better than that of group B, and the complication rate of group A (1 case) was significantly less than that of group B, with significant statistical difference.	Electroacupuncture combined with acupoint application can improve the clinical efficacy and reduce the incidence of complications in the treatment of facial neuritis, which is better than the electroacupuncture group.
Sha-bei Xu et al (2013)^[56]^	Patients diagnosed with facial neuritis (n = 338) Interventions: Patients were treated with prednisone and divided into acupuncture deqi, or acupuncture (control group).	Patients in the deqi group had better facial function (OR = 4.16, 95% CI: 2.23–7.78), better FDI assessment (LSM = 9.80, 95% CI: 6.29–13.30) and better quality of life (LSM = 29.86, 95% CI: 22.33–37.38); Deqi acupuncture had a positive effect on facial nerve function (OR = 1.07, 95% CI: 1.04–1.09).	In patients with facial neuritis, acupuncture treatment with strong stimulation of Deqi has a better effect, and the stronger the intensity of Deqi, the better the therapeutic effect.
Sinem Gökçe Kütük et al (2020)^[57]^	Patients diagnosed with facial neuritis (n = 88) Interventions: Patients received standard therapy, or standard therapy plus electroacupuncture.	The proportion of patients with facial neuritis treated with standard therapy combined with electroacupuncture who had electromyographically normal nerve function was significantly higher than those treated with standard therapy (66.7% vs 25.0%, *P* = .020); meanwhile, the proportion of patients with House-Brackmann grade ≤ 2 was higher (*P* < .05); Patients with a facial nerve recovery profile score of ≥ 8 were also significantly higher (*P* < .05) than those in the standard treatment group.	The results of the study suggest that standard therapy combined with electroacupuncture is superior to standard therapy alone in improving neurological dysfunction, reducing the severity of paralysis, and better functional recovery. Electroacupuncture may be a safe and promising adjunct to the treatment of facial neuritis.
Zhang Haiyan, et al (2021)^[58]^	Patients diagnosed with facial neuritis (n = 144) Interventions: The patients were randomly divided into control group (treated with Xiaoxumingtang placebo granules combined with acupuncture) and observation group (treated with Xiaoxumingtang combined with acupuncture).	The clinical efficacy of the observation group was better than that of the control group (*Z* = 2.014, *P* < .05). The score of FDI in the observation group was higher than that in the control group, and the score of wind-cold attacking collaterals was lower than that in the control group (*P* < .01). The scores of H-B rating scale and facial nerve function in the observation group were higher than those in the control group (*P* < .01).	Modified Xiaoxuming Decoction combined with acupuncture has good clinical efficacy in the treatment of acute peripheral facial paralysis (wind-cold attacking collaterals syndrome), and can regulate the immune function and inflammatory reaction of patients, with good safety.
Marten et al (2022)^[59]^	Patients diagnosed with facial neuritis (n = 73) Interventions: According to the principle of random, the patients were divided into the traditional Chinese medicine group (Siwu Decoction and Qianzheng Powder) and the acupuncture and medicine group (Siwu Decoction and Qianzheng Powder in the acute stage, combined with acupuncture treatment in the resting stage and recovery stage).	After 8 weeks of treatment, compared with the Chinese medicine group, the acupuncture and medicine group had a higher total effective rate after treatment; the acupuncture and medicine group had a more significant effect in improving House-Brackmann grade; the 2 groups of patients had a more significant effect on FDI physical function, social function score, and Chinese medicine symptom score of facial paralysis than the acupuncture and medicine group before treatment, and the above differences were statistically significant (*P* < .05).	Acupuncture and drug treatment of Bell’s palsy in stages can improve the curative effect, improve the symptoms of facial paralysis and facial nerve injury, and the advantages of acupuncture combined with drug treatment are obvious.
Jong-In Kim et al (2011)^[60]^	Six RCTs involved 512 patients diagnosed with facial neuritis; participants were treated with acupuncture in combination with medication or acupuncture.	Meta-analysis results showed a favorable effect of acupuncture on disease remission rates (RR = 1.11, 95% CI: 1.05 to 1.17; *P* = .001, I2 = 13%).	However, there is less evidence that acupuncture can be effective in treating facial neuritis. The number and quality of trials are too low to draw definitive conclusions.
Ziliang Zou. (2021)^[61]^	Seventeen RCTs involved 2644 patients diagnosed with facial neuritis; participants were divided into acute phase acupuncture treatment or acute phase without acupuncture treatment.	The results of meta-analysis showed that the cure time of facial neuritis treated with acupuncture in acute stage was shorter than that in non-acute stage (*P* < .05), and the incidence of sequelae of facial neuritis treated with acupuncture in acute stage was lower than that in non-acute stage (*P* < .05).	Compared with acupuncture in non-acute stage, acupuncture is safe and effective in the treatment of facial neuritis in acute stage, which shortens the cure time and reduces the occurrence of sequelae.
Li-Li Wang et al (2015)^[62]^	The 5-item RCTs involved 344 patients diagnosed with facial neuritis; participants were divided into acupuncture plus vitamin B12 acupoint injection or acupuncture alone.	The incomplete recovery rate of patients with facial neuritis was 44.50% in the acupuncture plus vitamin B12 group and 62.57% in the acupuncture alone group; acupuncture plus vitamin B12 was superior to acupuncture alone for Bell’s palsy (RR = 0.71, 95% CI: 0.58–0.87; *P* = .001).	Acupuncture combined with vitamin B12 may reduce the risk of incomplete recovery in patients with facial neuritis compared with acupuncture alone. This conclusion is uncertain due to study bias and methodological limitations.
Yunpeng Bian et al (2016)^[63]^	A total of 17 patients with facial neuritis were recruited for resting-state fMRI scans, which were re-scanned after standard acupuncture rehabilitation. Another 22 healthy controls were scanned once for comparison.	A comparison of FC before acupuncture between the healthy group and the patient group revealed an abnormal increase in FC in the patient group. At the same time, acupuncture can reduce FC in patients with facial neuritis. This study shows that FC modulation by acupuncture in patients with facial neuritis is specific and consistent with recovery trends.	This study provides new insights into treatment-related neural responses in patients with facial neuritis and suggests potential functional pathways for therapeutic evaluation in patients with facial neuritis. Future studies are still needed to confirm the current results and clarify the complex neural mechanisms of acupuncture treatment.

The first was a 2004 single-blind, multicenter, randomized controlled trial of 439 patients with facial neuritis that studied acupuncture and acupuncture plus medication for facial neuritis compared with medication. The results of the study showed that the combined treatment of acupuncture and moxibustion was superior to the drug treatment group, and the difference was statistically significant (C2 = 265.0, *P* = 018.2). The report did not count the adverse reactions of acupuncture and moxibustion, which was the regret of the trial. The study concluded that combination therapy with Acu-Moxi and acupuncture provides support as an effective alternative treatment for patients with facial neuritis.^[52]^

The second was a multicenter, large sample, single-blind, randomized controlled trial in 2006. The study included 480 patients diagnosed with facial neuritis in 4 hospitals, including the Affiliated Hospital of Chengdu University of Traditional Chinese Medicine, the First Affiliated Hospital of West China Medical Center of Sichuan University, Mianyang Hospital of Traditional Chinese Medicine and Sichuan People’s Hospital. The Patients with facial neuritis were randomly divided into 3 groups: control group (treated with prednisone, vitamin B1, vitamin B12 and bendazol), acupuncture group (treated with filiform needle and moxibustion) and basic treatment plus acupuncture group (control group plus acupuncture group). H-B rating scale and Facial Disability Index (FDI) were used to evaluate the efficacy after 4 weeks of treatment. After 4 weeks of treatment, it was found that the acupuncture group and the basic treatment plus acupuncture group were better than the control group in terms of clinical efficacy, Hourse-Branckmann score and the improvement of FDI score difference before and after treatment, with significant statistical difference. The results showed that the clinical efficacy of acupuncture combined with moxibustion and combined with basic treatment was better than that of single drug treatment, and the efficacy of acupuncture combined with drug treatment was definite, which was worthy of promotion.^[53]^

Thirdly, Lian-Sheng Yang et al^[54]^ conducted a retrospective analysis of 201 patients diagnosed with facial neuritis in 2020, by collecting patients with facial neuritis diagnosed and treated in the Third Affiliated Hospital of Sun Yat-sen University from 2016 to 2021. Patients were divided into 2 groups according to whether they had received initial acupuncture treatment within 7 days of the onset of paralysis. The acupuncture group (n = 76) received acupuncture within 7 days and the acupuncture group (n = 125) did not receive acupuncture within 7 days. The results showed that the time to complete recovery of symptoms was significantly shorter in patients with facial neuritis who received acupuncture within 7 days (HR = 1.505, 95% CI: 1.028–2.404, *P* < .05) compared with patients who did not receive acupuncture within 7 days. The group that received acupuncture within 7 days had a higher recovery rate at 12 weeks than the group that did not receive acupuncture within 7 days (93.4% vs 80.3%, *P* = .032). Studies have found that acupuncture combined with drug intervention in the acute stage of facial neuritis can shorten the recovery time of patients.

In a randomized controlled trial, 102 patients with Bell’s palsy were randomly divided into 2 groups: electroacupuncture combined with acupoint application of traditional Chinese medicine group (group a, 50 cases) and electroacupuncture group (group B, 52 cases). Electroacupuncture was used to treat Zanzhu (BL2), Yangbai (GB14), Taiyang (EX-HN5), Zygoma (SL18), Xiaguan (ST7), Yingxiang (LI20), etc; Yifeng (TE17) was treated with “No.1 Facial Paralysis.” The results showed that the scores of facial nerve function in group A and B were significantly improved after treatment (48.2 ± 2.9 vs 25.7 ± 4.9, 45.9 ± 6.2 vs 25.8 ± 5.5, *P* < .01), and the cure rate in group A [82.0% (41/50)] was significantly better than that in group B [67.0% (41/50)] (*P* < .01). The incidence of complications in group A (UNK21 cases) was significantly lower than that in group B (UNK48 cases), *P* < 0. 05. Compared with electroacupuncture, electroacupuncture combined with acupoint application can not only improve the clinical efficacy, but also reduce the incidence of complications.^[55]^

A 2013 RCT evaluated facial nerve function, sequelae, and quality of life at 6 months in patients with facial neuritis treated with Deqi acupuncture versus direct acupuncture without any manipulation (as a control). The authors found that patients in the deqi group had better facial function (OR = 4.16, 95% CI: 2.23–7.78), better FDI assessment (LSM = 9.80, 95% CI: 6.29–13.30), and better quality of life (LSM = 29.86, 95% CI: 22.33–37.38); At the same time, acupuncture and moxibustion had a positive effect on facial nerve function (OR = 1.07, 95% CI: 1.04–1.09). Therefore, the author concludes that acupuncture treatment with strong stimulation of Deqi has a better therapeutic effect, and the stronger the intensity of Deqi, the better the therapeutic effect.^[56]^

Sinem G Gökçe K Kütük et al studied electroacupuncture in combination with standard therapy in patients with facial neuritis, comparing outcomes such as clinical efficacy and neurophysiology. Participants in the study were divided into 2 groups: standard treatment group and electroacupuncture combined with standard treatment group. The aim of this study was to compare the 2 groups of patients with facial neuritis by evaluating the H-B grading system and the facial nerve recovery profile and electromyography before and after 12 weeks of treatment. The authors found that patients with facial neuritis treated with electroacupuncture plus standard therapy had a significantly higher rate of normal nerve function in EMG at week 12 compared with the standard therapy group (66.7% vs 25.0%, *P* = .020), and the rate of patients with H-B grade ≤ 2 was significantly higher at week 3 (79.2% vs 45.0%, *P* = .029), week 6 (87.5% vs 45.0%, *P* = .004), and week 12 (95.8% vs 50.0%, *P* = .001), and the rate of patients with a facial nerve recovery profile score ≥ 8 at week 6 of treatment (83.3% vs 45.0%, *P* = .011) and week 12 (87.5% vs 50.0%, *P* = .009). Therefore, the authors concluded that EA in combination with standard treatment was significantly superior to standard treatment alone in improving neurologic dysfunction, reducing the severity of paralysis, and better functional recovery in patients with facial neuritis. This seems to indicate that electroacupuncture may be a safe and promising adjunct in the treatment of facial neuritis.^[57]^

A prospective randomized controlled trial of acupuncture as a treatment option for facial neuritis was conducted in 2021. The treatment of facial neuritis was compared between acupuncture combined with traditional Chinese medicine and acupuncture combined with traditional Chinese medicine in order to determine the efficacy of acupuncture combined with traditional Chinese medicine in the treatment of facial neuritis. The authors reported that the scores of H-B facial nerve function grading scale, facial nerve function grading assessment, FDI scale and score of wind-cold attacking collaterals before treatment, 2 weeks and 4 weeks after treatment in acupuncture combined with Xiaoxuming Decoction were higher than those in acupuncture combined with Xiaoxuming Decoction and placebo (*P* < .01); At the same time, the levels of CD3+, CD4+, Treg and CD4+/CD8+ in the patients with acupuncture combined with Xiaoxuming Decoction were higher than those in the patients with acupuncture combined with Xiaoxuming Decoction placebo, while the levels of CD8+, Th17, Th17/Treg, TNF-α, IL-1β, and IL-6 were lower than those in the patients with acupuncture combined with Xiaoxuming Decoction placebo. Acupuncture combined with Xiaoxuming Decoction can inhibit the expression of proinflammatory factors, reduce inflammatory injury, and promote the balance of immune regulation. Therefore, the authors conclude that the combination of acupuncture and medicine can improve the facial nerve function defect of patients with facial neuritis, reduce the facial disability index and symptoms of traditional Chinese medicine, improve the quality of life, and regulate immune function and inflammatory response, with good clinical efficacy. However, this study is an exploratory, small sample, single-center study, and the mechanism of peripheral facial paralysis is unclear and complex, so the extrapolation of research conclusions has certain limitations.^[58]^

In 2022, a randomized controlled trial was conducted to explore the clinical efficacy of acupuncture and traditional Chinese medicine in the treatment of facial neuritis. Seventy-three patients with facial neuritis were randomly divided into Chinese medicine group (oral administration of modified Siwu Decoction and Qianzheng Powder) and acupuncture and medicine group (oral administration of modified Siwu Decoction and Qianzheng Powder at acute stage, combined with acupuncture at resting and convalescent stages). By observing the 2 groups of patients before and after treatment of facial nerve function classification, FDI, facial paralysis TCM symptom score, the curative effect was compared. The author’s study found that after treatment, the H-B classification, FDI index and TCM symptom score of the 2 groups were significantly improved compared with those before treatment (*P* < .05), and the improvement effect of the acupuncture and medicine group was more significant (*P* < .05).^[59]^

A meta-analysis of 6 trials involving 512 patients reported a favorable effect of acupuncture plus medication on disease remission rates compared to medication alone with the following parameters. The authors conclude that there is limited evidence to support the efficacy of acupuncture in the treatment of facial neuritis, and that the number and quality of trials are too low to draw definitive conclusions.^[60]^

Another meta-analysis examined 17 trials, including 2644 participants, to compare the efficacy and safety of acupuncture in the acute versus non-acute phase of facial neuritis. The primary endpoint of this meta-analysis was the efficacy and safety of facial neuritis. The analysis showed that the cure time of facial neuritis treated with acupuncture and moxibustion in the acute stage was shorter than that in the non-acute stage (*P* < .05), and the incidence of sequelae of facial neuritis treated with acupuncture and moxibustion in the acute stage was lower than that in the non-acute stage (*P* < .05). The authors concluded that acupuncture and moxibustion treatment of facial neuritis in acute stage was safe and effective compared with acupuncture and moxibustion treatment in non-acute stage, which shortened the healing time and reduced the occurrence of sequelae.^[61]^

A meta-analysis evaluated the efficacy of acupuncture combined with vitamin B12 acupoint injection compared with acupuncture alone in reducing incomplete recovery in patients with Bell’s palsy. The study included 5 trials involving a total of 344 participants. Analysis found that the results showed that the incomplete recovery rate of patients with facial neuritis was 44.50% in the acupuncture combined with vitamin B12 group and 62.57% in the acupuncture alone group. The combined effect size showed that acupuncture plus vitamin B12 was superior to acupuncture alone for Bell’s palsy (RR = 0.71, 95% CI: 0.58–0.87; *P* = .001). Most of the included studies were of medium or low quality and biased. The authors conclude that for patients with facial neuritis, acupuncture combined with vitamin B12 may reduce the risk of incomplete recovery compared with acupuncture alone.^[62]^

Finally, a 2016 study performed a between-group analysis of healthy controls and patients before acupuncture by comparing the activation mapping and facial movement-related regions of healthy controls with those of patients with facial neuritis to extract FC with altered onset time. Patients with facial neuritis after and before acupuncture were then analyzed between groups to find changes in FC mediated by acupuncture. The results showed that selective acupuncture could reduce the abnormally increased functional connectivity in patients with facial neuritis. These data suggest that acupuncture modulation of FC in patients with facial neuritis is specific and consistent with the recovery trend, and is beneficial to the recovery of the disease.^[63]^

## 4. Conclusion

Acupuncture is a traditional Chinese medicine therapy that uses stimulation at specific locations (points) on the patient’s skin to achieve therapeutic results. According to the clinical research literature in recent years, acupuncture and moxibustion treatment, as a mature treatment method, has a definite effect on facial neuritis and is widely recognized. Its treatment methods are diverse and have the advantages of low cost, simplicity and less adverse reactions. With the development and deepening of research, acupuncture and moxibustion treatment has gradually formed a comprehensive therapy of acupuncture and moxibustion combined with drugs from a single acupuncture treatment, which effectively improves the efficacy of acupuncture and moxibustion or single drug treatment of facial neuritis, and significantly improves the quality of life of patients with facial neuritis.

Modern research is difficult to elucidate the pathophysiology of facial neuritis, and there are various pathogenic hypotheses. At present, steroids, antiviral and neurotrophic drugs are the main clinical drugs, which have a long treatment cycle and side effects. However, acupuncture can significantly shorten the treatment period of patients with facial neuritis and reduce their clinical symptoms faster than drug treatment, which can be evaluated and found by researchers through clinical studies. Therefore, more and more attention has been paid to acupuncture and moxibustion as a supplementary means for the treatment of patients with facial neuritis, and the combined treatment of acupuncture and medicine for patients with facial neuritis is also a major trend in the future, which can give full play to the advantages of traditional Chinese and Western medicine treatment.

Many studies have shown that the combination of acupuncture and medication is a safe, useful, and available therapy for patients with facial neuritis. Importantly, our summary results also showed that the cure time and the incidence of sequelae in the combined acupuncture and medication group were lower than those in the simple medication group, which further indicated that the combined acupuncture and medication had certain advantages in the treatment of facial neuritis, and the application of combined acupuncture and medication in the treatment of facial neuritis should be further promoted.

However, more relevant basic research, animal experiments and large-scale clinical observations are needed to explain and clarify the strategies of traditional Chinese medicine in the treatment of facial neuritis, to further consolidate these findings, to provide further support for the clinical value of acupuncture and drug combination therapy, and to ensure that the inclusion of acupuncture and drug combination therapy in the treatment of facial neuritis can produce positive results for patients. In conclusion, substantial evidence from human studies supports a beneficial effect of acupuncture in patients with facial neuritis. However, the efficacy of combined acupuncture and drug therapy remains to be evaluated in large and well-designed clinical trials.

## Author contributions

**Conceptualization:** Qingxi Cao.

**Data curation:** Qingxi Cao, Lingyan Zhai.

**Formal analysis:** Qingxi Cao.

**Funding acquisition:** Qingxi Cao, Biao Qi.

**Investigation:** Qingxi Cao, Biao Qi.

**Methodology:** Qingxi Cao, Biao Qi, Lingyan Zhai.

**Project administration:** Qingxi Cao, Lingyan Zhai.

**Resources:** Qingxi Cao.

**Software:** Qingxi Cao.

**Supervision:** Qingxi Cao.

**Validation:** Qingxi Cao.

**Visualization:** Qingxi Cao.

**Writing – original draft:** Qingxi Cao, Lingyan Zhai.

**Writing – review & editing:** Qingxi Cao.
